# Abnormal urinary excretion of NKCC2 and AQP2 in response to hypertonic saline in chronic kidney disease: *an intervention study in patients with chronic kidney disease and healthy controls*

**DOI:** 10.1186/1471-2369-15-101

**Published:** 2014-06-26

**Authors:** Janni M Jensen, Frank H Mose, Anna-Ewa O Kulik, Jesper N Bech, Robert A Fenton, Erling B Pedersen

**Affiliations:** 1 Department of Medical Research, Holstebro Hospital, University Clinic in Nephrology and Hypertension, Laegaardvej 12, 7500 Holstebro, Denmark; 2 Regional Hospital Jutland West and Aarhus University, Aarhus, Denmark; 3 Department of Biomedicine, Aarhus University, Aarhus, Denmark

**Keywords:** Chronic kidney disease, NKCC2, AQP2, ENaC, Sodium, Water

## Abstract

**Background:**

Renal handling of sodium and water is abnormal in chronic kidney disease (CKD). The aim of this study was to test the hypothesis that abnormal activity of the aquaporin-2 water channels (AQP2), the sodium-potassium-2chloride transporter (NKCC2) and/or the epithelial sodium channels (ENaC) contribute to this phenomenon.

**Methods:**

23 patients with CKD and 24 healthy controls at baseline and after 3% saline infusion were compared. The following measurements were performed: urinary concentrations of AQP2 (u-AQP2), NKCC2 (u-NKCC2), ENaC (u-ENaCγ), glomerular filtration rate (GFR) estimated by ^51^Cr-EDTA clearance, free water clearance (C_H2O_), urinary output (UO), fractional excretion of sodium (FE_Na_), plasma concentrations of AVP, renin (PRC), Angiotensin II (ANG II), Aldosterone (Aldo) and body fluid volumes.

**Results:**

At baseline, GFR was 34 ml/min in CKD patients and 89 ml/ml in controls. There were no significant differences in u-AQP2, u-NKCC2 or u-ENaCγ, but FE_Na_, p-Aldo and p-AVP were higher in CKD patients than controls. In response to hypertonic saline, patients with CKD had an attenuated decrease in C_H2O_ and UO. A greater increase in U-AQP2 was observed in CKD patients compared to controls. Furthermore, u-NKCC2 increased in CKD patients, whereas u-NKCC2 decreased in controls. Body fluid volumes did not significantly differ.

**Conclusions:**

In response to hypertonic saline, u-NKCC2 increased, suggesting an increased sodium reabsorption via NKCC2 in patients with CKD. U-AQP2 increased more in CKD patients, despite an attenuated decrease in C_H2O_. Thus, though high levels of p-AVP and p-Aldo, the kidneys can only partly compensate and counteract acute volume expansion due to a defective tubular response.

**Trial registration:**

Clinical trial no: NCT01623661. Date of trial registration: 18.06.2012.

## Background

In chronic kidney disease (CKD), total renal mass is reduced and single nephron glomerular filtration rate increases in the remaining nephrons [1]. This hyper filtration, accompanied by a compensatory renal hypertrophy, are associated with alterations in tubular absorptive capacities of sodium and water that ultimately leads to an impaired ability to concentrate urine [2,3]. Animal studies have demonstrated that compared to healthy control animals, aquaporin-2 water channels (AQP2) and various sodium transporters are differentially expressed in rats with chronic renal failure (CRF) [4]. Furthermore, AQP2 has been linked to numerous conditions with disturbed water balance, including CKD [5-9]. Mutations in sodium transporters and their regulatory factors have been linked to disturbances in sodium homeostasis and blood pressure [10-12]. However, there is little knowledge of the involvement of these proteins in the defective urine concentrating mechanism in patients with CKD.

Quantification of the urinary excretion of the sodium-potassium-2-chloride cotransporter (NKCC2), AQP2 and the epithelial sodium channels (ENaC), as an estimate of their tubular abundance and thus their function, may provide important information regarding a dysfunctional transport activity in CKD [13-15]. Therefore, in the present study we performed an intervention study in patients with CKD stage III-IV and healthy control subjects during baseline conditions and in response to an acute 3% hypertonic saline infusion to test the following hypothesis; 1) the tubular handling of sodium and water is different in patients with CKD compared to healthy subjects and 2) Different sodium and water handling by the kidney is reflected by varying changes in urinary excretion of AQP2, NKCC2 and/or ENaC. Our study documents that in both healthy subjects and patients with CKD there are changes in u-NKCC2, in response to a hypertonic saline load.

## Methods

### Design

The study was conducted as an intervention study with a case group, consisting of patients with CKD, and a healthy control group. The CKD patients were matched with healthy controls one to one according to gender and age. An age discrepancy of three years was accepted.

### Recruitment

Patients with CKD were recruited from the Outpatient Clinic of Nephrology and Hypertension, Holstebro, Regional Hospital Jutland West. Healthy participants were recruited by advertisements in local newspapers, in the area of Holstebro, Denmark.

### Study settings

The study took place at Dept. of Medical Research, University Clinic of Nephrology and Hypertension, Regional Hospital Holstebro, Denmark, from 1^st^ of October 2011 until 1^st^ of May 2013.

### Participants

#### *CKD Patients*

*The inclusion criteria* for patients were: men and women; aged between 18 and 70 years; body mass index (BMI) ≤ 32 kg/m^2^; a diagnosis of CKD and estimated GFR (e-GFR) between 15–60 ml/min i.e. CKD stage III-IV. *The exclusion criteria* were: heart failure; pulmonary-, liver-, endocrine-, cerebral or malignant disease; immunosuppressive treatment; alcohol abuse (more than 21 alcoholic drinks per week for males and 14 drinks for females); difficulty in urinating; medicine abuse; currently smoking; pregnancy; unwillingness to participate. *Withdrawal criteria:* Development of one or more of the conditions given in ‘exclusion criteria’ during the course of the study; withdrawal of informed consent; poor compliance; problems with blood or urine sampling.

#### *Healthy control subjects*

*The inclusion criteria* for healthy controls were: men and women; aged between 18 – 70 years; BMI ≤ 32 kg/m^2^. *The exclusion criteria were:* clinical signs or history of heart-, pulmonary-, kidney-, cerebral-, endocrine or malignant diseases; abnormal findings in ECG, urine dipstick or biochemistry (blood cell count, b-hemoglobin, plasma concentrations of sodium, potassium, creatinine, albumin, glucose, bilirubin, alanine aminotransferase, alkaline phosphatase and cholesterol); arterial hypertension (24 hour-ambulatory blood pressure >130/80 mmHg); medical treatment (except oral contraceptives); alcohol abuse (more than 21 alcoholic drinks per week for males and 14 drinks for females); substance abuse; current smoking; pregnancy; breast feeding; donation of blood within one month prior to the study; unwillingness to participate. *The withdrawal criteria*: Development of one or more of the conditions given in exclusion criteria during the course of the experiment; withdrawal of informed consent; poor compliance; problems with blood or urine sampling.

### Ethics

This study was approved by the Regional Committee on Health Research Ethics (j. no. M-20110131) and carried out in accordance with the Helsinki Declaration. Written informed consent was obtained from all subjects.

### Effect variables

The main effect variable was u-NKCC2. Other effect variables were: u-AQP2, u-ENaCγ, glomerular filtration rate (GFR), free water clearance (C_H2O_), urine output (UO), fractional excretion of sodium (FE_Na_) and potassium (FE_K_), urine osmolality (u-osm), plasma sodium (p-Na), plasma osmolality (p-osm), plasma concentration of renin (PRC), angiotensin II (AngII), aldosterone (Aldo), vasopressin (AVP), extracellular fluid volume (ECV), intracellular fluid volume (ICV) and total body water (TBW).

### Number of subjects

Using a significance level of 5% and a power of 80% it was calculated that the number of subjects needed in both groups was 16, when the minimal relevant difference in u-NKCC2 was 0.3 ng/min and SD was 0.3 ng/min. In this study, incomplete voiding during study days was expected in some subjects; therefore a *minimum* of 20 subjects was included in each group.

### Experimental procedures

#### *Experimental procedure prior to the study day*

Four days prior to each study day, subjects consumed a standardized diet regarding calories, sodium and fluid. The diet consisted of 11,000 (kJ/day) with an energy distribution of 55% carbohydrates, 30% fat and 15% protein in accordance to general dietary guidelines. The sodium content was 130–150 mmol pr. day. The subjects were asked to drink 2500 ml/day. No alcohol or soft drink consumption was allowed while on the standardized diet. A maximum of two cups (6 oz.) of coffee or tea was allowed daily. Subjects were instructed to keep their usual physical activity during the experiments but to abstain from hard training the day prior to the examination. CKD patients followed their normal medical prescriptions during the four-day-diet period, but not on the morning of the study day. A 24-hour urine collection, ending at 7:00 AM on the examination-day, was used to assess water and sodium balance.

#### *Experimental procedure on the study day*

Following an overnight fast, subjects arrived at the study facility at 8:00 AM. Two indwelling catheters for blood sampling and administration of ^51^Cr-EDTA and saline were placed in both cubital veins. Every 30 minutes starting at arrival, participants received a 175 ml oral water load of tap water. Urine was collected in standing or sitting position. Otherwise, subjects were kept in the supine position in a quiet temperature-controlled room (22-25°C). At 9:00 AM a priming dose of ^51^Cr-EDTA was administered, followed by sustained infusion. Three 30-minute baseline clearance periods were obtained from 9:30 AM to 11:00 AM (time: 0–90 min). These were followed by one clearance period from 11:00 AM to 12:00 PM (time: 90–150 min) during which a sustained infusion of 3% hypertonic saline was administered. The post infusion period consisted of three 30-minute periods from 12:00 PM to 1:30 PM (time: 150–240 min).

Blood and urine samples were collected every 30 minutes from 8:30 AM to 1:30 PM. Blood samples were drawn and analyzed for ^51^Cr-EDTA, p-sodium, p-potassium, p-albumin and p-osmolality. Analysis of PRC, p-Ang II, p-Aldo and p-AVP were conducted from blood samples drawn at 11:00 AM, 12:00 PM and 1:30 PM.

Urine samples were analyzed for u-^51^Cr-EDTA, u-sodium, u-potassium, u-creatinine and u-osmolality. Analysis of u-AQP2, u-NKCC2 and u-ENaCγ was conducted from the 24-h urine collection and clearance period 10:30–11:00 AM (basal); 11:00–12:00 AM (cessation of fluid infusion), 12:00–12:30 PM (30 min after cessation of fluid infusion) and 1:00–1:30 PM (90 min after cessation of fluid infusion). For data analysis, the 30-minute periods from 9:30 AM to 1:30 PM were subdivided into: baseline (0–90 min), infusion period (90–150 min) and post infusion period (1:150–180 min, 2:180–210 min and 3: 210–240 min).

Body composition was measured at 8:30 AM (arrival), 11:00 AM (before infusion), 12:00 PM (after infusion) and 1:30 PM (end of examination day).

### Measurements

#### *Renal function*

Glomerular filtration rate was measured by the constant infusion clearance technique with ^51^Cr-EDTA as reference substance. More than 15% variation in GFR between the three baseline periods led to the exclusion of analysis.

#### *Calculations*

Fractional excretion of sodium and potassium was calculated as: [Sodium/potassium clearance (*C*_Na/K_)/GFR × 100%]. Free water clearance was calculated as: [Urine output (UO) – osmolar clearance (*C*_OMS_)]. *C*_OSM_ was calculated as: [Urine osmolarity/plasma osmolarity × UO].

#### *Blood samples*

Were centrifuged for 10 minutes at 2200 × g at 4°C. Plasma hormone samples were kept frozen at -20°C (AngII) and -80°C (PRC, Aldo, and AVP) until assayed. *Renin* in plasma was determined using an immunoradiometric assay (CIS Bio International, Gif-Sur-Yvette Cedex, France). Minimal detection level was 1 pg./mL the coefficients of variation were 14.5% (interassay) and 4.5% (intra assay). *Aldosterone* in plasma was determined by radioimmunoassay (Demeditec Diagnostics Systems Laboratories Inc.,Webster, TX, USA). Minimal detection level was 22 pmol/L. The coefficients of variation were 8.2% (inter-assay) and 3.9% (intra-assay). *Arginine vasopressin* and *Angiotensin II* were extracted from plasma with C_18_ Sep-Pak (Water associates, Milford, MA, USA) and subsequently measured using radioimmunoassay as previously described [16]. The antibody against angiotensin II was obtained from the Department of Clinical Physiology, Glostrup Hospital, Glostrup, Denmark. Minimal detection level was 2 pmol/L. The coefficients of variation were 12% (inter-assay) and 8% (intra-assay). The antibody against AVP was a gift from Professor Jacques Dürr (Miami, FL, USA). Minimal detection level was 0.2 pmol/L. The coefficients of variation were 13% (inter-assay) and 9% (intra –assay).

#### *Generation of NKCC2 specific antibody*

A novel rabbit polyclonal antiserum against human NKCC2 (*Slc12a2*) was generated against the following peptide: CNITKTTPKKDGSIN by Genscript® (New Jersey, USA). The N-terminal cysteine was added for conjugation to carrier protein and for attaching the peptide to the affinity purification column. The immune serum from two rabbits (#593 and #594) was affinity purified using immunizing peptides, resulting in NKCC2-specific antibodies.

#### *Urine sample immunoassays*

Urines were stored frozen at -20°C until assayed.

**U-NKCC2** was measured in urine by a newly developed radioimmunoassay. Iodination of the NKCC2 peptide was performed by the chloramine T method using 40 μg of 98% pure NKCC2 peptide and 37 MBq ^125^I. The reaction was stopped by the addition of 20% human serum albumin. ^125^I-labelled NKCC2 was separated from the iodination mixture by the use of a Sephadex G-25 Fine column. The assay buffer was 40 mM sodium phosphate (pH = 7.4), 0.2% human albumin, 0.1% Triton X-100 and 0.4% EDTA. A 1.5% solution of bovine gamma globulins (USB corporation, Cleveland, OH, USA) and 25% polyethylene glycol 6000 (Merck) also containing 0.625% Tween 20 (Merck) was prepared using the 40 mM phosphate buffer. After thawing, urine samples were centrifuged for 10 min at 2200 g (3500 rpm). A quantity of the supernatant containing 300 mosmol/l was freeze dried and stored at -20°C until assayed. Standards or freeze-dried urine samples were dissolved in 300 μL assay buffer with 0.2% human albumin, 50 μL of antibody was added, and the mixture was incubated for 24 h at 4°C. Thereafter, 50 μL of the tracer were added, and the mixture was incubated for a further 24 h at 4°C. Bovine gamma globulin (100 μL) and 2 mL polyethylene glycol 6000 were added. The mixture was centrifuged at 3500 rpm for 20 min at 4°C. The supernatant (free fraction) was poured off, and the precipitate (bound fraction) was counted in a gamma counter. The unknown content in urine extracts was read from a standard curve. For 9 consecutive standard curves, the zero standard was 35 ± 4.7%, and for increasing amounts of NKCC2 standard, the binding inhibition was: 33 ± 4.7% (0.0625 ng/tube), 32 ± 4.4% (0.125 ng/tube), 29 ± 3.9% (0.25 ng/tube), 25 ± 3.4% (0.5 ng/tube), 20 ± 2.8% (1.0 ng/tube), 15 ± 1.8% (2.0 ng/tube), 12 ± 1.5% (4.0 ng/tube), 10 ± 1.4% (8.0 ng/tube). The ID 50, i.e. the concentration of standard needed for 50% binding inhibition, was 1.37 ± 0.14 ng/tube (n = 9). Non-specific binding, determined by performing the RIA without antibody, was 6.7 ± 1.2% (n = 9).

*Inter-assay variation* was determined by quality controls from the same urine pool spiked with NKCC2 standard. In consecutive assays, the coefficient of variation was: at a mean level of 0.286 ng/tube 16% (14 assays), at a mean level of 0.736 ng/tube 12% (18 assays), at a mean level of 2.16 ng/tube 13% (15 assays), and at a mean level of 4.51 ng/tube 15% (14 assays).

*Intra-assay variation* was determined on samples from the same urine pool in several assays at different concentration levels. At a mean level of 0.126 ng/tube (n = 10) and 0.309 ng/tube (n = 10), the coefficients of variation were 7.5% and 6.0%, respectively. In addition, the coefficients of variation were calculated on the basis of duplicate determinations in different assays to 2.0% (n = 14) in the range 0.286-0.320 ng/tube, 5.9% (n = 18) in the range 0.731-0.736 ng/tube, 7.0% (n = 15) in the range 0.216-0.217 ng/tube, 7.3% (n = 14) in the range 0.451-0.468 ng/tube, and 1.3% (n = 61) in the whole range 0.286-0.468 ng/tube.

*Sensitivity* calculated as the smallest detectable difference at the 95% confidence limit was 0.71 ng/tube in the range 0.286–0.320 pg/tube (n =14), 0.13 ng/tube in the range 0.731-0.735 ng/tube (n = 18), 0.44 ng/tube in the range 2.16-2.17 ng/tube (n = 15), 1.04 ng/tube in the range 4.51-4.68 ng/tube (n = 14) and 2.35 ng/tube in the whole range 0.29-4.68 ng/tube (n = 61). The lower detectable limit of the assay was 0.5 ng/tube. It was calculated using the average zero binding for 9 consecutive assays minus 2 SD. The volume of urine used for extraction from the same pool was varied (17 different volumes in the range 250–5000 μl), and the mean concentration measured was 0.463 ± 0.066 ng/ml. There was a highly significant correlation between the extracted volume of urine and the amount of ng/tube (r = 0.979, n = 17, p <0.000). When NKCC2 in the range of 0.37-2.38 ng was added to urine, a significant correlation was found between the measured and the expected values (r = 0.896, n = 9, P < 0.001).

**U-AQP2** was measured by radioimmunoassay as previously described [17,18]. Antibodies were raised in rabbits to a synthetic peptide corresponding to the 15 COOH-terminal amino acids in human AQP2 to which was added an NH_2_-terminal cysteine for conjugation and affinity purification. Minimal detection level was 34 pg/tube /tube. The coefficients of variation were 11.7% (inter-assay) and 5.9% (intra-assay).

**U-ENaCγ** was measured by radioimmunoassay as previously described [19,20]. Antibodies were raised against a synthetic ENaCγ peptide in rabbits and affinity purified [21]. Minimal detection level was 48 pg/tube. The coefficients of variation were 14% (inter-assay) and 6.7% (intra-assay).

#### *Blood pressure measurement*

Brachial blood pressure was recorded using a semiautomatic oscillometric device (Omron 705IT, Omron Matsusaka, Japan)

#### *Plasma and urine*

Concentrations of sodium, potassium, creatinine and albumin were measured using routine methods at the Department of Clinical Biochemistry, Holstebro Hospital.

#### *Bioimpedance spectroscopy*

Was performed at 50 frequencies, from 5 to 1000 kHz using the Fresenius Body Composition Monitor and the Fluid Management Tool, version 3.

### Statistics

Statistical analyses were performed using IBM SPSS statistics version 20.0.0 (IBM Corp.; Armonk, NY, USA). Single baseline values were obtained by taking the weighed average of the measurements from the three baseline periods. Parametric data are presented as means ± standard deviation (SD) and nonparametric data as medians with 25th and 75th percentiles in brackets. General linear model with repeated measures (GLM RM) was performed for comparison with time as within-subject factor and group as between-subject factor, to test for differences within and between groups. Independent samples t-test or Mann–Whitney U test were used for comparison of CKD patients vs. controls and CKD patients stage IV vs. CKD stage III. Paired t-test or related samples Wilcoxon Signed Rank test were used to compare baseline within groups to the following periods. Statistical significance was defined as p < 0.05 in all analyses.

## Results

### Demographics

#### *CKD Patients*

**Figure 1:**
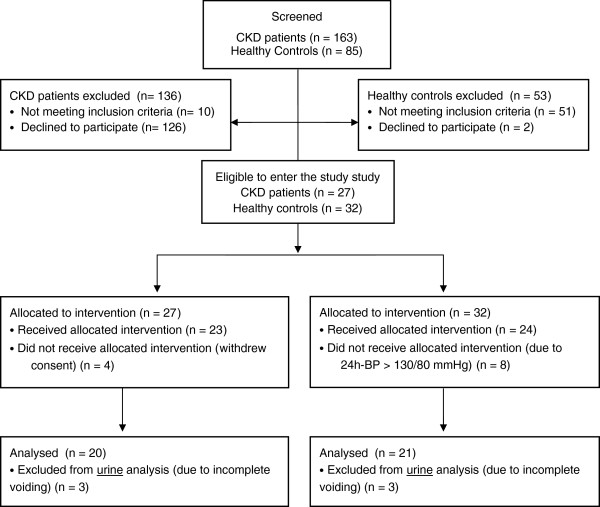
CONSORT flow chart over the participant flow through screening, inclusion and analysis.

Patients in the Outpatient Clinic were consecutively screened for participants to the study. Twenty-seven CKD patients were included in the study. Four patients withdrew consent. Thus, 23 CKD patients were initially allocated to, and completed the study. Three patients were not able to void satisfactorily during clearance experiments and were excluded from urine analyses (Figure 1).

The primary renal disease was nephrosclerosis due to essential hypertension (n = 7), chronic glomerulonephritis (n = 3), adult polycystic kidney disease (n = 3), interstitial nephropathy (n = 1), systemic sclerosis (n = 1) and unknown nephropathy (n = 8). All CKD patients received antihypertensive treatment in various combinations i.e. angiotensin converting enzyme inhibitor (n = 12), calcium channel antagonists (n = 12), loop diuretics (n = 11), beta-adrenergic blockers (n = 9), thiazides (n = 7), angiotensin II receptor antagonists (n = 6), potassium sparing diuretics (n = 1) and minoxidil (n = 1).

#### *Healthy controls*

A total of 32 subjects were screened to enter the study, but eight subjects were withdrawn due to arterial hypertension. Thus, 24 healthy controls were initially allocated to and completed the study. Three subjects were not able to void satisfactorily during clearance experiments and were excluded from urine analysis only (Figure 1).

Baseline characteristics are shown in Table 1. Values were similar between the two groups with the exception of plasma creatinine (p-Crea), eGFR, plasma urea, urine albumin (u-Alb), p-potassium and p-hemoglobin.

**Table 1:** Baseline characteristics and 24-hour urine collection in 23 patients with CKD and 24 healthy controls

	**Patients (n = 23)**	**Healthy Controls (n = 24)**	***p *** **value**
Gender, n (Female/Male) (%)	6 (26)/17 (74)	6 (25)/18 (75)	
Age (years)			
Males	60 ± 10	58 ± 9	0.52
Females	63 ± 8	60 ± 8	0.53
Body Mass Index (kg/m^2^)			
Males	27.4 ± 2.6	25.6 ± 2.2	0.03
Females	24.4 ± 4.4	23.2 ± 1.6	0.54
24-h BP (mmHg)	124/76 ± 15/8	123/74 ± 7/5	0.72/0.25
Pulse Rate (min^-1^)	68 ± 10	65 ± 8	0.19
Screening Biochemistry			
p-sodium (mmol/l)	141 ± 2	142 ± 2	0.22
p-potassium (mmol/l)	4.4 ± 0.6	4.0 ± 0.3	0.02
p-albumin (g/l)	43 ± 3	43 ± 2	0.34
p-hemoglobin (mmol/l)	8.4 ± 0.7	8.9 ± 0.7	0.01
p-creatinine (μmol/l))	199.7 ± 81.6	76.7 ± 12.0	<0.0001
eGFR (ml/min)	31 ± 15	87 ± 15	<0.0001
p-urea (mmol/l)	12.7 ± 4.9	5.1 ± 1.2	<0.0001
u-albumin (mg/l)	8 (5; 65)	5 (2; 8)	0.01
**24-h urine collection**			
Urine Output (ml/24 h)	2741 ± 753	2667 ± 817	0.75
u-osm (mosm/24 h)	831 ± 219	917 ± 150	0.12
C_H2O_ (ml/min)	-0.029 ± 0.52	-0.355 ± 0.56	0.04
Creatinin Clearance ml/min * m^2^	46 ± 21	114 ± 17	<0.0001
u-AQP2 (ng/mmol)	144 ± 29	139 ± 23	0.55
u-ENaCγ (ng/mmol)	96 ± 41	90 ± 33	0.55
u-NKCC2 (ng/mmol)	377 ± 99	365 ± 98	0.70
u-Na (μmol/min)	99 ± 37	94 ± 32	0.63
FE_Na_ (%)	1.71 ± 0.99	0.53 ± 0.19	<0.0001
u-K (μmol/min)	47 (37; 53)	50 (46; 59)	0.11
FE_K_ (%)	21 (16; 28)	11 (9; 12)	<0.0001

Values are means ± SD. U-albumin is the median with 25^th^ and 75^th^ percentiles in brackets.

BP, Blood pressure; eGFR, estimated glomerular filtration rate. Urine osmolality (u-osm), free water clearance (C_H2O_), urinary excretion of AQP2 (u-AQP2), ENaCγ (u-ENaCγ) and NKCC2 (u-NKCC2) corrected for creatinin, urinary sodium excretion rate (u-Na), fractional excretion of sodium (FE_Na_), urinary potassium excretion rate (u-K) and fractional excretion of potassium (FE_K_).

*P* values in right column represent the probability of a difference between groups.

#### *NKCC2 antibody characterization*

**Figure 2:**
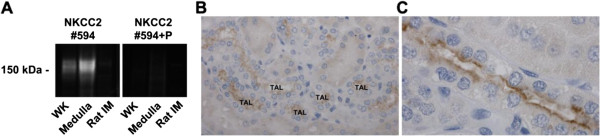
**NKCC2 antibody characterization. A)** Western blotting using a novel human NKCC2 specific antibody. The affinity-purified antibody (#594), detected a glycosylated smear characteristic of NKCC2 in human whole kidney (WK) and medulla samples, but not in rat inner medulla (IM). This signal was not observed following pre-incubation of the antibody with the immunizing peptide (P). **B)** Immunohistochemistry of a human kidney section using the novel NKCC2 antibody. Labeling was confined to the TAL. **C)** At higher magnification, the majority of NKCC2 signal was detected at the apical pole of TAL cells.

The affinity-purified antibodies were utilized for western blotting of human kidney samples using standard procedures. Both antibodies detected a glycosylated smear of approximately 170 kDa representing NKCC2 in cortex and medulla (Figure 2A), but no signal in a rat inner medulla protein sample. The specific protein bands were not observed following pre-incubation of the antibody with the immunizing peptide. Immunohistochemistry of human kidney sections demonstrated strong labeling at the apical pole of thick ascending limb cells (Figure 2B,C). No labeling of similar sections was detected following pre-incubation of the antibody with the immunizing peptide (not shown).

#### *Twenty-four-hour urine collection*

Table 1 shows the results of the 24-h urine collection. Mean u-AQP2, u-NKCC2, u-ENaCγ, urinary sodium-, potassium-, osmolarity- and volume did not differ significantly. Controls had a small, but significantly more negative C_H2O_ and higher creatinine clearance than patients with CKD. Both FE_Na_ and FE_K_ were significantly higher in the CKD patient group. No significant differences in u-NKCC2 were detected between patients treated with furosemide vs. no-furosemide in 24-h urine collection (406 ng/mmol ± 108 vs. 357 ± 92; p = 0.293) or during the examination day (data not shown).

#### *Excretion of water and u-AQP2, urine osmolality and GFR*

**Figure 3:**
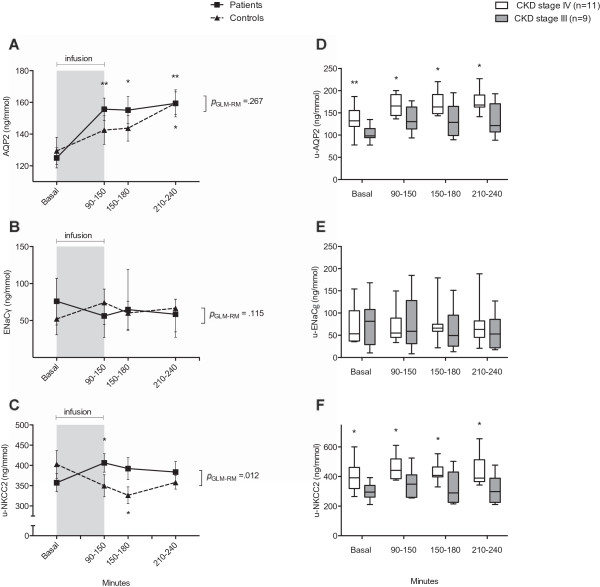
**Effects of 3% hypertonic saline on urinary excretion of A) AQP2, B) ENaCγ and C) NKCC2 in 20 patients with CKD and 21 healthy controls, and (D-F) u-AQP2, u-ENaCγ and u-NKCC2 in 20 patients classified according to GFR in CKD stage IV (n = 11) and CKD stage III (n = 9). A**-**C**: General linear model (GLM-RM) did not show any difference in u-AQP2 or u-ENaCγ but there was a significant difference in u-NKCC2. Values of u-AQP2 and u-NKCC2 are means ± SEM, u-ENaCγ is median with 25th and 75th percentiles. Paired t-test was used to test for differences between post-infusion to baseline. *p < 0.001; **p < 0.0001. **D**-**F**: Independent samples t-test showed a significant difference between CKD stage IV vs. stage III in u-AQP2 and u-NKCC2. Values are medians with 2.5th and 97.5th percentiles. *p < 0.05; **p < 0.0001.

**Figure 4:**
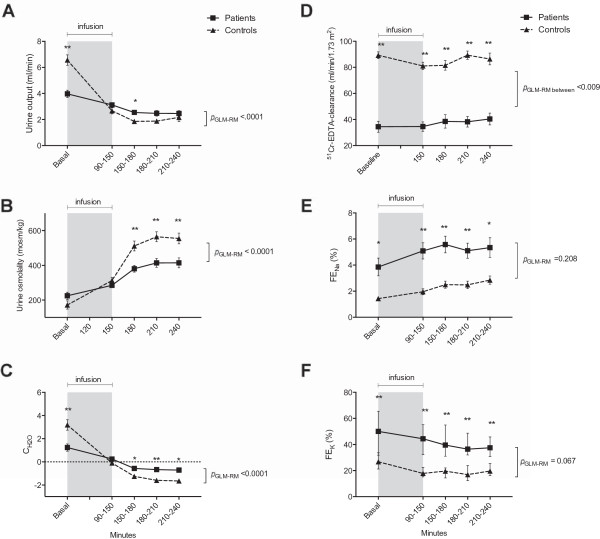
**Effects of 3% hypertonic saline on (A) Urine output, (B) Urine osmolality, (C) Free water clearance, (D) **^**51**^**Cr-EDTA-clearance, (E) FE**_**Na **_**and (F) FE**_**K **_**in 20 patients with CKD and 21 healthy controls.** Values of ^51^Cr-EDTA-clearance, UO, u-osm, CH2O and FE_Na_ are means ± SEM. FE_K_ is median with 25th and 75th percentiles. **A**-**C**: General linear model (GLM) with repeated measures was significant in both OU, u-osm and C_H2O_. **D**:P_GLM-RM_ between groups was significant. There was a significant response to hypertonic saline as GFR decreased in healthy controls, but increased in CKD patients **E**-**F**: P_GLM-RM_ were not significant in FE_Na_ and FE_K_, indicating that the response to 3% saline were similar between groups. Independent-samples t-test or Man Whitney U test in CKD patients vs. controls *p = 0.01, **p < 0.0001

Figure 3A shows the changes in u-AQP2, Figure 4A-D the changes in, UO, u-osm, C_H2O_ and GFR.There was no difference in u-AQP2 at baseline (Figure 3A). At the end of hypertonic saline infusion, u-AQP2 increased significantly in CKD patients but not in controls. During the post infusion periods, u-AQP2 gradually increased in controls, whereas u-AQP2 reached a plateau in CKD patients. By the end of the examination day, there was no difference between the groups.

GFR decreased in controls during the infusion period and reached baseline levels one hour after infusion had ceased. In CKD patients, however, GFR was unchanged during the infusion period after which GFR increased towards the end of the study day.

Mean baseline levels of UO were lower in the patient group than in controls. In both groups, the hypertonic saline induced a significant reduction. Initially, UO decreased less in CKD patients than in controls {(mean difference at 150 min patients: -15% ± 31 vs. controls: -56% ± 17), *p =* 0.03}. At the end of the examination, the relative decrease in UO in CKD patients was significantly lower than controls {(mean difference at 240 min patients: -29% ± 39 vs. controls: -64% ± 25), *p =* 0.02}.

At baseline u-osm were higher in patients than controls, but there was no significant difference. U-osm increased significantly in both groups during 3% hypertonic saline, but to a higher extent in controls than in patients {(mean difference at 240 min patients: 289 ± 155% vs. mean difference controls: 125 ± 141%), *p =* 0.001}.

At baseline, C_H2O_ was lower in the CKD patient group compared to controls and decreased significantly in both groups, but less in CKD patients. Both groups changed from positive values at baseline to negative values after infusion indicating a change from free water excretion to water reabsorption. C_H2O_ remained higher in CKD patients compared to controls throughout the examination day, but the relative change did not differ significantly {(mean difference at 240 min: patients: -132% ± 122 vs. controls: -155% ± 55), *p =* 0.099}.

#### *Excretion of sodium, potassium, u-NKCC2 and u-ENaCγ*

Figure 3B and 3C shows the change in u-ENaCγ and u-NKCC2. Figure 4E-F shows the change in FE_Na_, and FE_K._during the study day. At baseline FE_Na_ and FE_K_ were higher in CKD patients compared to controls. After hypertonic saline, FE_Na_ increased significantly and FE_K_ decreased significantly in both groups.

There was no difference in u-NKCC2 at baseline between CKD patients and controls. However, u-NKCC2 increased significantly in the CKD patient group and decreased significantly in the control group, in response to hypertonic saline infusion. At the end of examination, there was no difference between the two groups. However, the relative change in u-NKCC2 was significant higher in CKD patients than controls {(mean difference at 240 min patients: 10% ± 28 vs. controls: -10% ± 27), *p =* 0.028}.

At baseline, u-ENaCγ was higher in CKD patients, but the difference was not significant. During the examination day, there was a general trend towards a reduction in u-ENaCγ in CKD patients and an increase in controls, but it was not statistically significant. Nor were there a difference in the relative changes in u-ENaCγ between CKD patients and controls throughout the examination day.

#### *High u-AQP2 and u-NKCC2 in patients with kidney failure stage IV*

We performed a post hoc sub analysis of u-NKCC2, uAQP2 and u-ENaCγ dividing patients with CKD into GFR ≤ 30 ml/min/1.73 m^2^ i.e. stage IV (n = 11; mean GFR: 21 ml/min/1.73 m^2^) and GFR > 30 ml/min/1.73 m^2^ i.e. stage III (n = 9; mean GFR: 48 ml/min/1.73 m^2^) (Figure 3D-F).Patients with CKD stage IV had a higher u-AQP2, at baseline, than CKD stage III and u-AQP2 remained significantly higher throughout the examination day (Figure 3D). There were no detectable differences in u-ENaCγ either at baseline or after hypertonic saline infusion (Figure 3E). U-NKCC2 was significantly higher in patients with CKD stage IV than CKD stage III, both at baseline and in response to hypertonic saline infusion (Figure 3F).

### Vasoactive hormones

**Figure 5:**
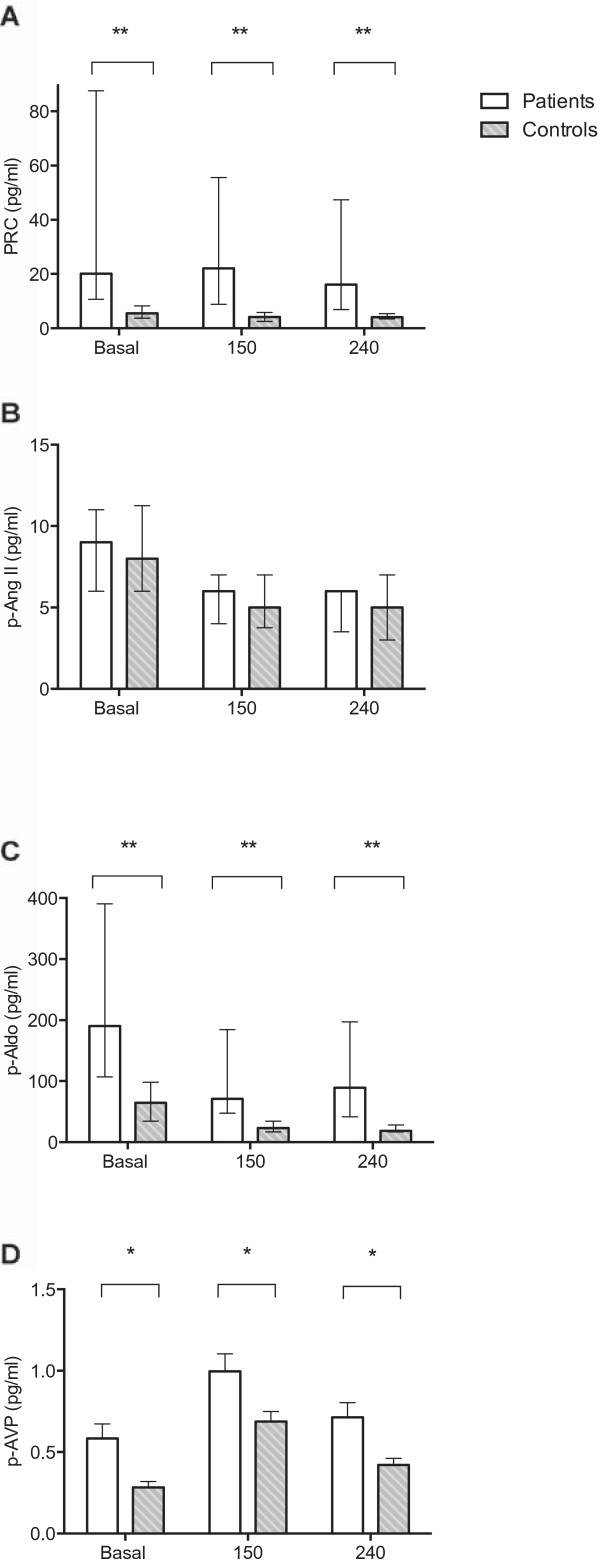
**Effects of 3% hypertonic saline on plasma concentrations of Renin (A), angiotensin II (B), aldosterone (C) and vasopressin (D).** PRC, p-ang II and p-aldo are presented as medians ±25th and 75th percentiles. P-AVP is mean ± SEM. **A**-**C**: Both groups decreased in response to hypertonic saline. PRC and p-Aldo were significant higher in the patient group. **D**: There was a significant difference in p-AVP between groups, but the response to hypertonic saline did not differ (pGLM-RM = 0.970). Independent-samples t-test or Man Whitney U test in CKD patients vs. controls *p = 0.01, **p < 0.0001.

Figure 5 shows PRC, p-Ang II, p-Aldo and p-AVP. At baseline, there were significantly higher levels of PRC and p-Aldo in CKD patients, but p-Ang II did not differ between groups. All three hormones were suppressed in response to hypertonic saline in both groups. PRC and p-Aldo levels remained significantly higher in CKD patients than controls after hypertonic saline infusion. One patient received spironolactone and elevated aldosterone. Exclusion of this subject from analysis had no impact on the overall results.

P-AVP was higher at baseline in CKD patients compared to controls. P-AVP increased significantly in both groups and to the same extent in response to hypertonic saline infusion, with a maximum at 150 min and a uniform steady reduction during the post infusion period. Dividing patients according to stage of CKD showed that p-AVP tended to be higher in CKD stage IV than CKD stage III, but there was no significant difference (data not shown).

### Plasma sodium, -potassium and -osmolality

At baseline, CKD patients had a significantly lower p-Na (p < 0.05). P-Na increased in response to hypertonic saline in both groups. The increase in p-Na was higher in patients compared to healthy controls at the end of the examination day, but it was not significant (patients: 2 ± 0.8% vs. controls: 1.6 ± 0.8%; p = 0.082). Patients with CKD had higher p-osm than controls (p < 0.0001). P-Osm increased to the same extent in both groups in response to hypertonic saline infusion. P-K was significant higher in the patient group throughout the examination day (p = 0.001), but did not change in response to hypertonic saline (p = 0.147) (data not shown).

### Bioimpedance spectroscopy

**Figure 6:**
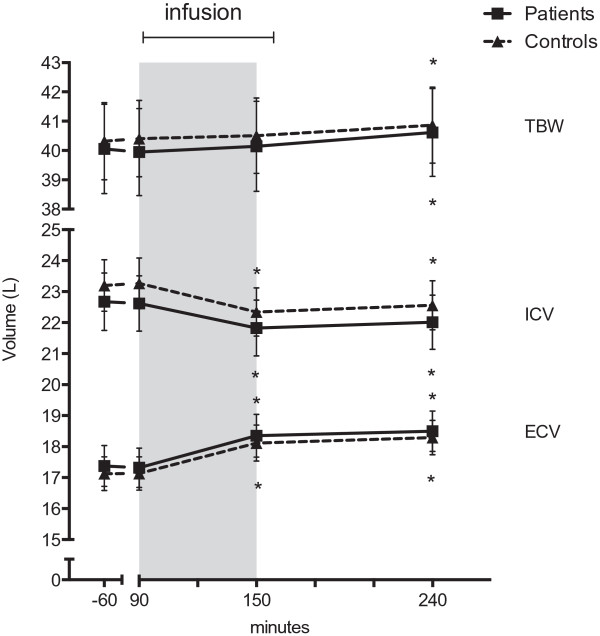
**Bioimpedance spectroscopy.** Effects of hypertonic saline infusion on total body water (TBW), intra cellular volume (ICV) and extracellular volume (ECV). Values are means ± SEM. *p < 0.0001 paired t-test infusion and post infusion periods vs. baseline.

Figure 6 shows the changes in TBW, ICV and ECV during the baseline period, infusion period and post infusion period.

The two groups received the same volume of hypertonic saline (patients: 557 ± 87 ml vs. controls: 533 ± 79 ml; p = 0.331) and an additional 1925 ml of tap water. As expected, when administering hypertonic saline, ICV decreased while ECV increased during the infusion period reaching a new steady state in the post infusion period. TBW also increased slightly and reached a maximum at the end of study day. Although there was a tendency towards a lower ICV and a higher ECV in CKD patients there were no statistically significant differences in the volume status between the patient and the control group. CKD patients also had a higher adipose tissue mass (ATM) compared to healthy controls {27.4 ± 9.4 kg vs. 32.1 ± 9.4 kg; p < 0.002}, but lean tissue mass (LTM) did not differ between the two groups {47.4 ± 9.7 kg vs. 47.9 ± 10.3 kg; p = 0.470}.

## Discussion

In the present study, we measured u-NKCC2, u-AQP2, and u-ENaCγ in CKD patients stage III-IV and healthy controls at baseline and in response to an intravenous volume load of 3.0% hypertonic saline in order to estimate and analyze sodium- and water channel function in patients with CKD compared to healthy controls. The novelty of this study is two-fold: 1) This is the first study that documents the changes in u-NKCC2 in healthy subjects as well in patients with CKD in response to a hypertonic saline load, 2) Measurements of the urinary excretion of NKCC2, AQP2 and ENaCγ in the same study has never been performed previously.

When compared to the patient group as a whole, there were no measurable differences in u-NKCC2, u-AQP2 and u-ENaCγ at baseline. In response to hypertonic saline, u-NKCC2 increased, u-AQP2 increased excessively and u-ENaC decreased in CKD patients compared to healthy controls. When patient groups were compared according to stage of CKD, there was a significant increase in u-NKCC2 and u-AQP2 in patients with CKD stage IV compared to CKD stage III, both at baseline and in response to hypertonic saline. This study further confirmed that patients with CKD stage III-IV showed a reduced renal concentrating ability compared to healthy subjects.

In this study, we used a novel and original method to evaluate sodium reabsorption in the ascending limb of Henles loop using a radioimmunoassay of urinary NKCC2. The quantity of excreted NKCC2 in urine is supposed to reflect the activity of sodium transport via NKCC2, just as u-AQP2 reflects the functional status of AQP2 water channels [9,18,22-24] and u-ENaCγ reflects the functional status of ENaC in the principal cells [20,25]. The signal that is being quantified is derived from specific antibody binding against peptides of NKCC2 as in AQP2 and ENaCγ - likely in urinary exosomes. Traditionally, tubular transporters are excreted in urinary exosomes, containing apical membrane and intracellular fluid [26,27]. Isobe et al. found an increased total and phosphorylated NCC in urinary exosomes in patients with PHAII and Lubbe et al. also found an increased number of phosphorylated NCC in urinary exosomes in patients with primary hyperaldosteronism [28,29]. Thus, there is evidence that the differences in transporters are due to higher protein abundance in the exosomes. It is well established that there is an increased trafficking of vesicles to the apical membrane upon stimulation [27,30,31]. However, it is unclear to what extent, there is an increased rate of shedding of the plasma membrane into exosomes.

Reabsorption of sodium via NKCC2 in the TAL is critical for the establishment of a hypertonic medullary interstitium and urine concentration [3,32,33]. In animals, AVP has been demonstrated to increase NKCC2 activity [34]. This effect is mediated by V2 receptors via Adenylate Cyclase 6, which facilitate phosphorylation and trafficking of NKCC2 to the apical membrane, thereby increasing NaCl reabsorption [31,34-36]. In humans, the regulation of NKCC2 is still unknown both under physiological as well as pathophysiological conditions. However, enhanced NaCl reabsorption via NKCC2 is associated with hypertension and decreased NaCl transport via NKCC2 results in low blood pressure, as seen in Bartter’s syndrome [12,37].

In response to hypertonic saline, a decrease in u-NKCC2 was observed in healthy controls, which is inconsistent with animal studies showing that AVP increases NKCC2 expression via a short-term mechanism [31,34]. However, as NKCC2 is associated to exosomes, which results from retrieval of plasma membrane proteins following agonist removal, there has to be a point where membrane NKCC2 increases before u-NKCC2 increases. Thus, due to a delayed response, a lack of change in u-NKCC2 does not necessarily reflect a lack of change within the plasma membrane of kidney epithelial cells. Therefor, AVP probably did play a role, but we did not see the full impact.

In patients with CKD, u-NKCC2 increased in response to hypertonic saline. When patients were grouped according to CKD stage IV and III, it was evident that patients with stage IV CKD had an abnormal increased excretion of NKCC2 at baseline and in response to hypertonic saline. There might be several explanations: Firstly, it seems feasible that fractional decreased proximal tubular sodium absorption might contribute to an exaggerated increase in sodium absorption via NKCC2 in CKD compared to healthy controls. Secondly, high p-AVP was apparent in CKD, and it has been reported that long-term elevated AVP up regulates the expression of NKCC2 to obtain maximal urinary concentration [34]. Thirdly, the increased u-NKCC2 in patients with CKD may be a compensatory phenomenon that reflects the need of more active sodium transport in the remaining thick ascending limbs to create a sufficient level of medullary hypertonicity to enhance water reabsorption via AQP2 along the collecting ducts.

U-AQP2 increased in both groups after hypertonic saline. Recently, and in agreement with the present study, our group reported an increase in u-AQP2 in response to hypertonic saline in healthy young subjects [38]. U-AQP2 increased excessively in CKD patients during the infusion period, and it was clearly illustrated in the post-hoc sub-analysis that patients with stage IV CKD had a significant higher u-AQP2, and thus an evidently abnormal response. Two previous studies have compared u-AQP2 in patients with CKD and diabetic nephropathy to healthy controls [9,39]. Both studies, however, found a reduced u-AQP2 in patients compared to controls. Firstly, in this study, the concentrating mechanism was manipulated by use of an acute volume expansion with hypertonic saline. In the previous studies, the urine concentrating mechanism was performed by 8–12 hours of water deprivation. The mechanisms involved might be different and explain the discrepancy in u-AQP2. Secondly, it has been demonstrated that fractional water reabsorption is reduced in the proximal nephron parts in CRF rats treated with volume expansion [40]. It is possible that a decrease in water reabsorption in proximal tubules is followed by a larger compensatory reabsorption of water in the collecting ducts of patients with CKD compared to healthy controls and patients with moderate renal impairment. Thirdly, as mentioned earlier, the increased u-NKCC2 in patients with CKD, may enhance medullary hypertonicity to increase water reabsorption via AQP2.

In response to hypertonic saline, UO and C_H2O_ decreased less pronounced in patients with CKD compared to controls. Thus, patients reabsorbed *less* water despite that patients exhibited higher u-AQP2 and p-AVP. Increased p-AVP, in CKD, might be compensatory to stimulate the remaining AQP2 water channels adequately, in order to concentrate urine. Indeed, increased p-AVP has been reported in rats with CRF and patients maintained on hemodialysis [41,42]. Although AVP increased the number of activated AQP2 water channels, it might not have been sufficient, to reabsorb the proper amount of water via AQP2. Thus, despite increased u-AQP2, C_H2O_ was less pronounced in patients compared to healthy controls, and thus concentration ability was compromised. Studies have indicated that defective urinary concentration ability may be an impairment of the AVP stimulated water reabsorption in the collecting ducts, either by an AVP resistant down regulation of AQP2 or down regulation of the V2 receptor protein [43-45]. To support this fact, there was no difference in baseline u-AQP2, but twice as high p-AVP in CKD patients compared to controls.

Thus, u-AQP2 was abnormal in CKD patients, and apparently more pronounced in patients with severe renal impairment. Perhaps due to an impaired response to AVP stimulation or a compensatory response to increased sodium reabsorption via NKCC2.

In the distal tubules, sodium transport occurs via the ENaC in the luminal membrane of principal cells [46]. P-aldo was increased by a factor three in CKD patients compared to controls. It is well known in CKD [47-49]. As aldosterone regulates ENaC, we initially expected a major difference in u-ENaCγ. However, aldosterone increases ENaC γ-cleavage and not its expression [50]. This might explain in part, why u-ENaCγ was not increased despite high p-aldo in CKD patients. However, due to the time lag of aldosterone’s action on sodium balance, it is unlikely that aldosterone had any major impact on the changes in u-ENaCγ during the study day. In CKD patients, we measured a decreased u-ENaCγ in response to hypertonic saline, indicating a decreased sodium reabsorption via ENaC. Apparently, and opposite healthy controls, CKD patients might decrease their sodium reabsorption via ENaC to compensate for an increased reabsorption via the NKCC2 channels. Thus, the decrease in u-ENaCγ in CKD patients and the increase in u-ENaCγ in healthy controls might be compensatory mechanisms due to an altered absorptive activity in NKCC2, but further studies are necessary to clarify this hypothesis..

### Estimation of body fluid volumes by bioimpedance spectroscopy

The determination of body fluid volumes via bioimpedance spectroscopy (BIS) is an accurate method for estimating total body water and the distribution of water between the intracellular and extracellular spaces [51]. In this present study, we measured no statistical difference between the two groups, but ICV was lower and ECV and TBW were increased in patients with CKD compared to controls. This is in agreement with previous findings [52,53]. We expected a more pronounced increase in TBW in patients with CKD, but the differences in ECV and ICV between CKD patients and controls were very small. There might have been a difference in body fluid volumes between CKD patients and healthy controls, but it was not detected in the present study, possibly due to the small number of subjects in each group.

### Strengths and limitations

The major strength of this study was the design as a case-controlled interventional study with an age and gender matched group of healthy controls. The test conditions were very well defined regarding diet, sodium and fluid intake. Thus, the results are not confounded by differences in intake of sodium and water. In addition, the study population was well defined, regarding renal function, and had moderately to severe reduced renal function. Although we found a significant difference in u-NKCC2 and u-AQP2 in stage IV CKD patients compared to CKD stage III, we must emphasize that it was a post-hoc analysis. This study was not conducted to demonstrate changes between CKD stages. Therefore the results are indicative and further studies must be performed to elucidate these findings.

Also, the causes of CKD were a mixture of primary diseases that may have hidden important information. We tested the handling of sodium and water in CKD in patients, who were receiving antihypertensive treatment, including diuretics, during the study period. Although the study was performed under standardized conditions, this could be a source of error as it may influence the excretion of sodium and components of RAAS. However, we tried to compensate for this by securing the subjects were in a fasting condition, and their morning prescription was postponed to the end of the examination day. We did not find it ethically justified to withdraw medication for a longer period. To overcome this, we could have compared CKD patients to a second control group of hypertensive patients with normal renal function but receiving similar therapy.

## Conclusions

After hypertonic saline, u-NKCC2 increased, in patients with CKD compared to healthy controls, thus indicating an increased sodium reabsorption via the NKCC2 transporter in CKD. U-AQP2 increased more in CKD patients, despite an attenuated decrease in C_H2O_ in CKD compared to healthy controls. Thus, the ability to concentrate urine was clearly impaired in patients with CKD.

In conclusion, though high levels of aldosteron and vasopressin, patients with CKD can only partly compensate and counteract an acute volume expansion with 3% hypertonic saline, due to a defective tubular function.

## Competing interests

The authors declare that they have no competing interests. The authors alone are responsible for the content and writing of this paper.

## Authors’ contributions

All authors have contributed to the manuscript. JMJ, FHM and EBP designed the project. JMJ, FHM and AEO performed the experiments and statistical analyzes. RAF performed the NKCC2 antibody characterization. JMJ, FHM, JNB, RAF and EBP wrote and edited the manuscript. All authors read and approved the final manuscript.

## Pre-publication history

The pre-publication history for this paper can be accessed here:

http://www.biomedcentral.com/1471-2369/15/101/prepub
